# Association between maternal folic acid and/or multivitamin supplementation time and fetal congenital heart disease: based on the China birth cohort study

**DOI:** 10.7150/ijms.102843

**Published:** 2025-01-01

**Authors:** Jingjing Wang, Simin Zhang, Lijuan Sun, Li Wang, Qingqing Wu

**Affiliations:** 1 Clinical Trial Institution Office, Beijing Obstetrics and Gynecology Hospital, Capital Medical University. Beijing Maternal and Child Health Care Hospital. Beijing 100026, China.; 2 Department of Ultrasound, Beijing Obstetrics and Gynecology Hospital, Capital Medical University. Beijing Maternal and Child Health Care Hospital. Beijing 100026, China.

**Keywords:** folate, multivitamin, congenital heart disease, pregnancy, fetal

## Abstract

**Background:** A multitude of studies have presented inconsistent outcomes regarding the association between maternal folic acid (FA) and/or multivitamin (MV) supplementation and congenital heart disease (CHD) in offspring. This study aimed to estimate supplementation time and CHD based on a prospective China birth cohort study (CBCS).

**Methods:** In the CBCS, 114,670 singleton pregnant women who had pregnancy outcomes until August 2021 and responded to the early pregnancy questionnaire were recruited. The participants were divided into three groups: no FA or MV supplementation, supplementation commencing before pregnancy, and supplementation commencing from early pregnancy. Unadjusted and adjusted logistic regression analyses were employed to calculate the odds ratio (OR) to estimate the relative risk (RR) value of CHD exposure to FA and/or MV. Additionally, the results of this study were combined with previous studies to calculate the pooled RR. Finally, stratification and sensitivity analyses, including the propensity score matching method, were conducted to identify the robustness of the association.

**Results:** Compared with the non-supplemented group, the RRs of CHD in groups with FA and/or MV supplementation, with supplementation before pregnancy, and with supplementation from early pregnancy were 1.23 (95% confidence interval [CI]: 0.76-2.00), 1.30 (95% CI: 0.80-2.13) and 1.19 (95% CI: 0.73-1.93), all demonstrating no statistically significant difference. The pooled RR from the forest plot was 0.98 (95% CI: 0.95-1.01), which is consistent with the findings of this study. Furthermore, the results remained approximately the same in the stratification or sensitivity analyses in different datasets, including performing 1:1 or 1:2 propensity score matching.

**Conclusions:** The present study suggests that FA or MV supplementation before or during early pregnancy may not influence the risk of offspring developing CHD.

## Background

Congenital heart disease (CHD) is a structural defect in the heart and great vessels that occurs before birth. It is the most prevalent congenital disability, affecting approximately one out of every 100 newborns, with an increasing global prevalence reported 1-3. The incidence of perinatal CHD in China has risen from 2000 to 2011, ranking first according to a report 4. Furthermore, a systematic review and meta-analysis of 617 studies revealed a continuous increase in CHD birth prevalence in China over time 5. The precise causes of CHD remain unknown. However, researchers have identified several factors that may contribute to the development of CHD 6, 7, and poor nutrition (e.g., low folic acid intake) may interact with genetics 8.

The supply of folate, an essential vitamin in mammals, depends entirely on the folate contained in dietary intake 9. Folic acid (FA) or multivitamins (MV) are taken during the first trimester to prevent fetal neural tube defects 10, but the role of its supplementation in preventing fetal CHD is controversial. In a systematic review and meta-analysis 11 that included six studies 12-17 examining the association between periconceptional use of MV and CHD, its relative risk (RR) was 0.83 (95% CI, 0.70-0.98), but after excluding the study with high risk of bias, a non-significant RR of 0.88 (95% CI, 0.77-1.01) was demonstrated. Another more recent meta-analysis 18 that included 37 studies with eight articles 19-26 published in 2017 and later suggested that periconceptional FA supplementation was associated with a decreased risk of CHD (RR= 0.79; CI: 0.71-0.89). Ito K et al. 27 analyzed 14,896 pregnant women enrolled from 2003 to 2012 at 13 weeks gestation, and the results showed no significant association between maternal serum folate in the first trimester and lower risk of any congenital disability or ventricular septal defect. A study 28 based on the China Birth Cohort Study (CBCS) 29 revealed that although excessive FA supplementation in pregnant women might increase the risk of birth defects in their offspring, the RR values of groups exposed only to FA, MV, or both FA and MV for CHD were not statistically significant. Furthermore, we should consider the association between the supplementation commencing time and CHD.

Considering MV supplementation contains FA and relatively few cases of CHD, this study explored the association between maternal FA and/or MV supplementation commencing time and the risk of CHD in offspring by classifying participants into three groups and using multivariable statistical models as well as matching methods that can adjust for potential confounding factors.

## Methods

### Study design and data sources

All data originated from the CBCS 29, a prospective, longitudinal, multi-center birth cohort study featuring three follow-up visits at mid-pregnancy, late pregnancy, and after delivery. From the initiation of the CBCS in November 2017 to August 2021, pregnant women who responded to the early pregnancy questionnaire and had fetal outcomes (n = 132,536) were retrieved from the electronic system of the CBCS.

**Figure 1:**
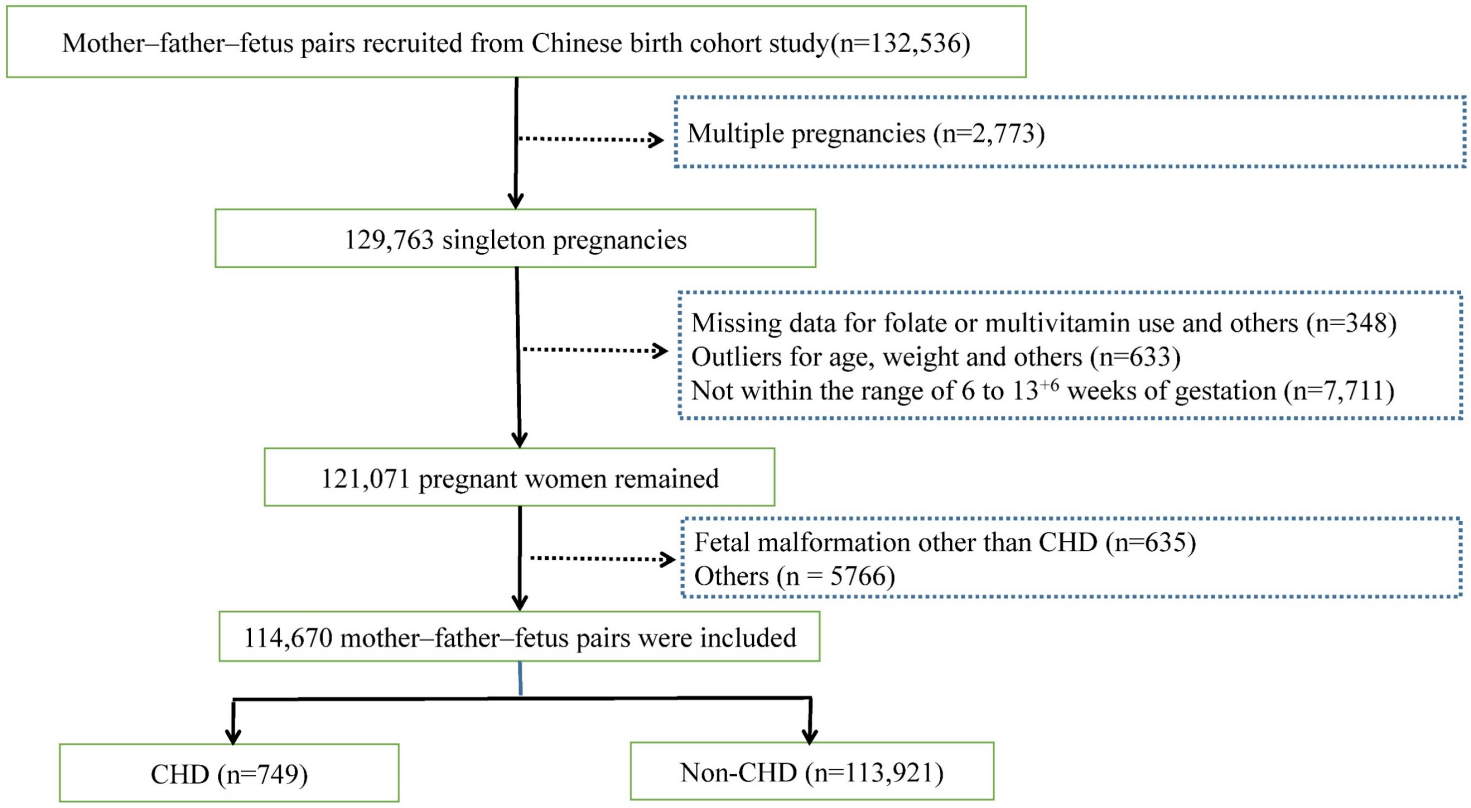
Flowchart of the study participant selection. Abbreviations: CHD: congenital heart disease.

The exclusion criteria encompassed multiple pregnancies (n = 2773), absence of data on FA or MV supplementation and other crucial values (n = 348), outliers (n = 633), and gestational age not within the range of 6 to 13 + 6 weeks (n = 7711). The rationales for these exclusions were as follows: 1) considering the impact of multiple pregnancies on nutritional requirements; 2) as for pregnant women filling out the early pregnancy questionnaire, the absence of data or outliers of such crucial information is not the accurate representation of themselves and might lead to bias. Additionally, we excluded abnormal conditions of maternal and fetal bodies other than fetal CHD (n = 6401, including fetal malformation, miscarriage, pregnancy loss, preterm birth, low birth weight, fetal macrosomia, etc.). Hence, 114,670 cases were ultimately included in accordance with the inclusion and exclusion criteria (Fig. 1).

The research was approved by the Ethics Committee of Beijing Obstetrics and Gynecology Hospital, Capital Medical University (Reference No. 2018-KY-003-02). Participation in the study was voluntary, and written informed consent was obtained.

### Data collection and measurements

In CBCS, women were enrolled during early pregnancy and completed the baseline questionnaire by themselves during this period. This study collected the following information from the CBCS: a) Demographic characteristics obtained from the questionnaire, including the investigation date, maternal birth date, weight, height, ethnicity, education, family income, and occupation. b) Current pregnancy information, including folic acid and multivitamin use in early or pre-pregnancy, the last menstrual period, conception mode, parity from the questionnaire, and pregnancy outcomes from the follow-up visits. c) Lifestyle behaviors obtained from the questionnaire, including maternal alcohol consumption, smoking, and secondhand smoke exposure. CHD diagnosis was extracted from the system without specific classification. All CHDs in the cohort were reported from the subcenters and evaluated by trained obstetricians, pediatricians, or cardiologists. Additionally, echocardiography, magnetic resonance imaging (MRI), or autopsy were conducted to determine suspected cases when appropriate. The early pregnancy questionnaires included two general inquiries on oral FA and MV supplementation for pregnant women. These inquiries are "When did you commence taking oral FA (such as Slian or other brands)?" and "When did you commence taking a MV (e.g., Alvy or other brands)?". The response options were three: none, before pregnancy, or during early pregnancy. Since MV supplementation also contains FA in this cohort, the FA and/or MV exposure status was classified into three groups: no FA or MV supplementation group, supplementation commencing before pregnancy group, and supplementation during early pregnancy group.

Other variables of this study are classified as follows: a) BMI was categorized into obesity (BMI ≥ 30.0 kg/m2), overweight (25.0-29.9 kg/m2), normal (18.5-24.9 kg/m2), and underweight (<18.5 kg/m2) in accordance with WHO criteria. d) Although such a classification would be controversial, occupations were divided into three categories based on previous papers 30: a) Active (e.g., farmer or manual laborer); b) Moderate (e.g., teacher, salesperson, or clerk); c) Light (e.g., unemployed).

### Statistical analysis

Firstly, the continuous data of the baseline characteristics among the three exposure groups were expressed as mean and standard deviation and tested by ANOVA for those with an approximately normal distribution or median and interquartile range and the Kruskal-Wallis H test for those without a normal distribution. Meanwhile, the categorical variables were depicted as frequency and percentage and tested by the Chi-square test or Fisher's exact test (when the conditions for the Chi-square test were not met) for nominal variables or the Kruskal-Wallis H test for ordinal data.

Secondly, in contrast to the group without FA or MV supplementation, the association between the supplementation groups commencing before or during pregnancy and the risk of offspring CHD was analyzed by undertaking unadjusted and maximally adjusted multivariate logistic regression (LR) analysis. Subsequently, the crude OR and adjusted OR (aOR) were calculated, which could offer an approximation of RR or aRR. Instead of log-binomial regression analysis, the LR method was implemented based on two considerations: (1) The OR derived from LR analysis can visually present the risk or protective effect and provide an approximation of RR. Although it might overestimate RR if the outcome is common, the proportion of CHD in this current study was relatively low, accounting for 0.7%. Thus, OR could be used to estimate RR in this study. (2) The data enlisted in this current study were selected after certain cases were deleted so that the calculated constituent ratio of CHD was not the actual incidence of CHD. The covariates selected were based on the existing variables in questionnaires, the P value of univariate analysis (less than 0.2), and expert advice. In addition, this study was combined with previous studies and mapped the random-effects meta-analysis forest plots.

Furthermore, stratification and sensitivity analyses were conducted to identify the robustness of the association. Stratification analyses were carried out in subgroups, including parental age, BMI status, ethnicity, educational level, physical activity, parity, drinking, smoking, family income, mode of conception, and gestational week. Sensitivity analyses were conducted in the following datasets: 1) dataset after excluding those CHD cases with genetic anomalies, 2) dataset after excluding those with other congenital disabilities, and 3) datasets after applying propensity score matching 31 that adjusts for unbalanced confounders by matching CHD and control subjects.

IBM SPSS Statistics version 22, SAS version 9.4, and STATA version 17.0 standard edition were employed to calculate and analyze data. A P value of less than 0.05 was regarded as statistically significant.

## Results

### Characteristics

Among all participants, 114,670 singleton pregnancies were ultimately included, of which 3199 (2.8%), 48,058 (41.9%), and 63,413 (55.3%) pregnant women were without FA or MV supplementation, FA and/or MV supplementation starting before pregnancy, and commencing from early pregnancy, respectively. Additionally, 749 (0.7%) had offspring with CHD. Table 1 indicated that maternal age, BMI status, ethnicity, educational level, physical activity, parity, drinking, smoking, family income, mode of conception, and gestational week were significantly different among those three supplementation groups (all *P* values < 0.05), while fetal CHD was not (*P* values = 0.06). In addition, the participants were also divided into groups according to their supplementation commencing time to FA (including none, pre-pregnancy, and early pregnancy) or MV (including none, pre-pregnancy, and early pregnancy) or supplementation types (including none, exposure to only FA, exposure to only MV, and exposure to FA and MV). The CHD rates in those groups are presented in Supplementary Figure A1.

### Maternal supplementation time to FA and/or MV and the risk of fetal CHD

Table 2 shows the results of non-adjusted and adjusted multivariable LR models. Compared with the group without FA or MV supplementation, the supplementation groups did not affect the risk of CHD in their offspring in the non-adjusted LR model. In adjusted LR model (adjusting age, BMI status, ethnicity, education level, physical activity, parity, drinking, smoking, family income, mode of conception, and gestational week), no effect was detected [FA and/or MV supplementation group (aOR=1.23; 95% CI, 0.76-2.00); supplementation before pregnancy group (aOR=1.30, 95% CI, 0.80-2.13); supplementation from early pregnancy group (aOR=1.19, 95% CI, 0.73-1.93)].

**Figure 2:**
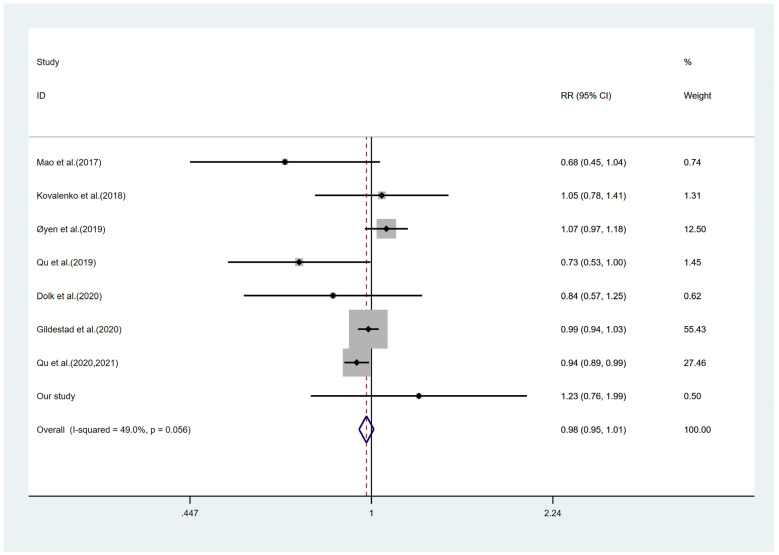
Forest plots of the effect estimates for congenital heart disease, including this study and the previous seven studies. The square size is proportional to the precision of the study-specific effect estimate, and the bar indicates the corresponding 95% CIs. The diamond is centered on the summary effect size of all included studies, and the width means the associated 95% CI. RR, risk ratio; CI, confidence interval

Besides, with reference to the latest relevant published meta-analyses and considering the variations in economic status over time, this study was combined with relative articles published since 2017 (Figure 2). After incorporating this study, the forest plots indicated similar results to this research (RR = 0.98; 95%CI: 0.95-1.01). Moreover, integrating this study's results into one of the earlier meta-analyses published in 2017^11^, the results revealed that maternal FA and/or MV supplementation significantly decreased the risk of CHD (RR = 0.87; 95%CI: 0.78-0.97). However, excluding the study with a high risk of bias suggested by that meta-analysis 11 led to a non-significant RR of 0.91 (95%CI: 0.82-1.02), similar to the previous 11 and this study.

### Stratification and sensitivity analyses

The association between maternal FA and/or MV supplementation time and the risk of fetal CHD did not demonstrate statistically significant differences across subgroups, as detailed in Table A1 in the Appendix. These subgroups were stratified by factors including age, BMI status, ethnicity, education level, physical activity, parity, drinking, smoking, family income, mode of conception and gestational week.

Furthermore, after excluding CHD cases with genetic anomalies (Dataset S1) or those additional congenital disabilities labeled in the dataset (Dataset S2), we obtained Dataset S1 and S2 comprising 733 and 712 cases of fetal CHD, respectively. The results from both unadjusted and adjusted multivariable LR models based on Dataset S1 and S2 indicated that maternal FA and/or MV supplementation commencing before or during early pregnancy did not influence the risk of CHD in offspring (Table 3).

**Figure 3:**
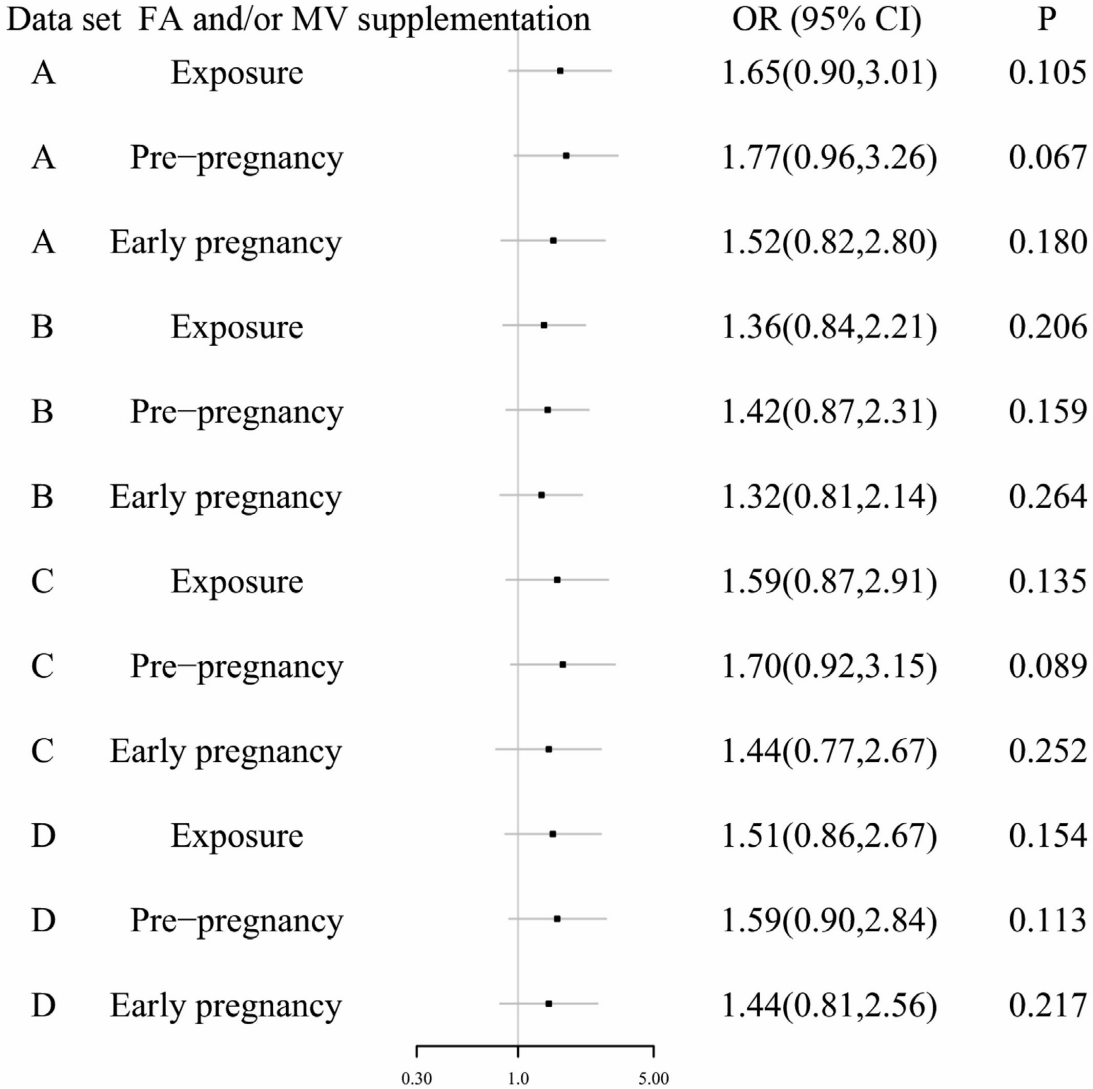
The results of conditional logistic regression analysis using datasets after propensity score matching methods. Dataset A contained 749 cases with fetal CHD and 749 normal cases. Dataset B contained 749 cases with fetal CHD and 1498 normal cases. Dataset C contained 712 cases with fetal CHD and 712 normal cases. Dataset D contained 712 cases with fetal CHD and 1424 normal cases. The group without maternal FA and MV supplement was used as the reference.

Additionally, propensity score matching methods at ratios of 1:1 and 1:2 were employed to comparatively evaluate the effects of maternal FA and/or MV supplementation time on the risk of fetal CHD. Consequently, Dataset A and Dataset B were generated containing 1498 and 2247 cases, respectively. Moreover, further application of propensity score matching methods at ratios of 1:1 and 1:2 was also conducted using Dataset S2, resulting in Dataset C and Dataset D, which included 1424 and 2136 cases, respectively. Conditional logistic regression analysis utilizing these four datasets, Dataset A through D, consistently revealed that compared to the group without FA and MV supplementation, maternal FA and/or MV supplementation initiated during early pregnancy or pre-pregnancy had no discernible impact on offspring's risk for developing (Figure 3).

## Discussion

### Main findings of this study

In this multicenter investigation utilizing the largest birth cohort in China, maternal FA and/or MV supplementation prior to or during early pregnancy does not appear to influence the risk of CHD in their offspring. The results remained robust even after incorporating our study into recently published literature or conducting stratification and sensitivity analyses in different datasets.

### Possible reasons and comparison with previous studies

In recent years, the effectiveness of FA supplementation in preventing CHD has garnered significant research attention. A case-control study involving 197 mothers of offspring with CHD and 788 matched unaffected mothers from Shanghai Province in China indicated that elevated maternal red blood cell folate levels were associated with a reduced risk of CHD 32. Another study 33 employing a 1:2 matched case-control design, which involved 1800 cases from Shanxi Province in China over two years (2014 - 2016), demonstrated that FA supplementation during pregnancy was associated with a decreased risk of fetal CHD (OR=0.60, 95%CI: 0.45-0.82). Furthermore, a multivariate analysis 34 based on data from a tertiary referral hospital revealed an independent association between CHD and FA and/or MV use during the first trimester (RR=0.69, 95% CI: 0.55-0.87). However, some studies have reported no association between FA or MV intake and CHD in offspring 21, 22, 24, 25, 28, leaving the protective effect of FA and/or MV supplementation against CHD inconclusive.

The frequently discussed mechanism involves homocysteine being converted back to methionine through reactions by folate and vitamin B12, elevated homocysteine levels are linked to deficiencies in active forms of folate and B12^9^, ^35^. Improper usage of vitamin supplements can lead to adverse health effects. For instance, one study 36 indicated that nicotinamide riboside, a form of vitamin B3, could elevate the risk of triple-negative breast cancer if not used properly and could cause cancer to spread to the brain.

Gianforcaro et al. 37 conducted a retrospective cohort study involving 445 fetuses with CHD and genetic testing, revealing that genetic abnormalities were identified in 29.4% of the pregnancies. Given that these fetal genetic anomalies or other defects could potentially influence this association between maternal FA and/or MV supplementation and fetal CHD, sensitivity analyses were performed on datasets excluding such cases, yielding consistent results. However, it is essential to acknowledge that this study only excluded cases with genetic test results or congenital disabilities already documented in the dataset when exporting from the CBCS system. Additionally, there were instances of subsequent abnormal test results not recorded or lacking genetic testing within both the CHD and normal groups. These factors represent the limitations of this research.

One plausible explanation for why maternal FA and/or MV supplementation during early pregnancy or preconception did not significantly affect the risk of CHD in offspring may be attributed to economic development influences. This investigation was based on a Chinese cohort from 2017 to 2021 29, during which time the living standard for pregnant women improved alongside more nutritious diets; consequently, any benefit derived from additional FA or MV usage might be diminished. Dolk et al. 22 found no protective effect associated with periconceptional FA supplementation but noted a protective effect linked to frequent consumption of folate-rich fruits. Pregnant women enrolled in this study who did not utilize FA or MV supplements may have maintained a nutritionally balanced diet. Pregnant women may not suffer from nutritional deficiency due to the nonuse of FA or MV supplements before or during pregnancy without suffering deficiencies due to their nonuse before or during pregnancy. Research addressing similar topics in less developed regions may yield divergent outcomes; unfortunately, dietary intake for pregnant women throughout gestation was not included in this analysis.

Another consideration is that maternal exposure to FA alone generally consists of 0.4 mg per day, while over 60% of participants received a dose exceeding this amount, as reported by CBCS data. Furthermore, those exposed to MV also ingested various vitamins, including vitamin A, vitamin D, iron, and zinc, which may be potentially associated with the risk of CHD in offspring. Additionally, adverse effects stemming from elevated folate levels or poor absorption rates could further complicate the associations observed 28. It is also possible that after completing the questionnaires, pregnant women, especially among high-risk individuals, pregnant women become more vigilant about nutritional supplements during subsequent pregnancies.

As widely recognized, numerous factors contribute to the incidence of 6, 7. The information on susceptible genotypes needs to be included in this study. Webber et al. show that FA supplementation is associated with a lower risk of CHD in families with specific risk alleles, including risk alleles for folic acid metabolism 38. Despite conducting multivariate analyses, stratified analyses, sensitivity analyses after propensity score matching methods, or combined with previous studies to minimize confounding, bias remains a possibility, thus emphasizing the necessity for comprehensive surveys assessing perinatal maternal nutrient intake as well as randomized controlled trials moving forward.

### Strengths and limitations of the study

Our study possesses several strengths. Firstly, it draws an extensive dataset sourced from a Chinese cohort, allowing us insight into correlations between maternal FA or MV supplementation time and the risk of fetal CHD. Secondly, we performed multivariate analysis, stratified and sensitivity analysis, and integrated our findings with previous studies to verify the robustness of the results.

Nonetheless, several limitations must be acknowledged:

a) As previously mentioned, due to the relatively small number of CHD cases and the absence of specific subcategories, we only included some confounding factors, making it impossible to conduct further analysis of a particular type of CHD.

b) In this study, we deleted records with missing data on MV and FA supplements or other crucial variables instead of a multiple imputation, which may result in bias.

c) The cohort explored the potential risk factors for fetal congenital disabilities. It focused on the role of maternal FA and MV supplementation through only two relatively simple questions. Additionally, it lacks information on the susceptible genotypes, and information on genetic abnormalities is incomplete. Moreover, only a relatively low proportion (2.8%) of women did not take FA and MV supplements. All those may cause biased estimates and suggest that replication in other large populations is necessary.

## Conclusions

Our research indicates that initiating maternal FA or MV supplementation prior to or during the early stages of pregnancy does not appear influential regarding subsequent risks associated with the offspring's development into having CHD. However, that is not to say that FA or MV supplementation does not affect the prevention of fetal congenital disabilities. Strategies to prevent congenital disabilities through FA supplementation in pregnant women need further validation, especially for offspring with CHD. A full-term perinatal nutritional intake survey or randomized controlled trial is still required.

## Supplementary Material

Supplementary figure and table.

## Figures and Tables

**Table 1:** Characteristics of participants in different groups

Groups	*n*	FA and/or MV supplementation (*n*/%)			*P* values
		None	Pre-pregnancy	Early pregnancy	
Maternal age (years)					<0.001
<35	99,897	2624 (2.6)	42,169 (42.2)	55,104 (55.2)	
≥35	14,773	575 (3.9)	5889 (39.9)	8309 (56.2)	
Paternal age (years)					<0.001
<35	90,390	2348 (2.6)	38,371(42.5)	49,671 (55.0)	
≥35	24,280	851 (3.5)	9687 (39.9)	13,742 (56.6)	
BMI before pregnancy					<0.001
Normal (18.5-24.9)	83,487	2239 (2.7)	36,075 (43.2)	45,173 (54.1)	
Low (<18.5)	15,754	407 (2.6)	6045 (38.4)	9302 (59.0)	
Overweight (25.0-29.9)	12,581	415 (3.3)	4964 (39.5)	7202 (57.2)	
Obesity (≥30.0)	2848	138 (4.8)	974 (34.2)	1736 (61.0)	
Ethnicity					<0.001
Han	107,927	2946 (2.7)	45,217 (41.9)	59,764 (55.4)	
Minority	6744	253 (3.8)	2841 (42.1)	3649 (54.1)	
Family income (CNY/year)					<0.001
<200,000	78,069	2481 (3.2)	30,962 (39.7)	44,626 (57.2)	
≥200,000	36,601	718 (2.0)	17,096 (46.7)	18,787 (51.3)	
Educational level					<0.001
College/university	79,000	1803 (2.3)	34,449 (43.6)	42,748 (54.1)	
High school or below	23,930	1239 (5.2)	7180 (30.0)	15,511 (64.8)	
Postgraduate	11,740	157 (1.3)	6429 (54.8)	5154 (43.9)	
Physical activity					<0.001
Moderate	53,740	1429 (2.7)	22,918 (42.6)	29,393 (54.7)	
Light	27,950	1115 (4.0)	10,205 (36.5)	16,630 (59.5)	
Active	32,980	655 (2.0)	14,935 (45.3)	17,390 (52.7)	
Parity					<0.001
Multipara	59,114	2312 (3.9)	20,797 (35.2)	36,005 (60.9)	
Nullipara	55,556	887 (1.6)	27,261 (49.1)	27,408 (49.3)	
Smoking before or during pregnancy					<0.001
No	112,132	3126 (2.8)	47,243 (42.1)	61,763 (55.1)	
Yes	2538	73 (2.9)	815 (32.1)	1650 (65.0)	
Secondhand smoke exposure					<0.001
No	101,612	2696 (2.7)	43,576 (42.9)	55,340 (54.5)	
Yes	13,058	503 (3.9)	4482 (34.3)	8073 (61.8)	
Drinking before or during pregnancy					<0.001
No	110,962	3086 (2.8)	46,908 (42.3)	60,968 (54.9)	
Yes	3708	113 (3.0)	1150 (31.0)	2445 (65.9)	
Mode of conception					<0.001
Natural conception	110,247	3171 (2.9)	44,933 (40.8)	62,143 (56.4)	
Assisted reproduction	4423	28 (0.6)	3125 (70.7)	1270 (28.7)	
Fetal CHD					0.060
No	113,921	3182 (2.8)	47,713 (41.9)	63,026 (55.3)	
Yes	749	17 (2.3)	345 (46.1)	387 (51.7)	
Gestational week (weeks)					<0.001
6~8	31,786	1092 (3.4)	15,441 (48.6)	15,253 (48.0)	
9~10	20,468	540 (2.6)	8332 (40.7)	11,596 (56.7)	
11~14	62,416	1567 (2.5)	24,285 (38.9)	36,564 (58.6)	

Abbreviations: BMI, body mass index; CHD, congenital heart disease; FA, folic acid; MV, multivitamins

**Table 2:** Associations of FA and/or MV supplementation on fetal CHD

FA and/or MV supplementation	Non-adjusted LR model OR (95% CI)	Adjusted LR model aOR (95% CI)
None	Ref	Ref
Supplementation	1.23 (0.76-1.99)	1.23 (0.76-2.00)
Pre-pregnancy	1.35 (0.83-2.25)	1.30 (0.80-2.13)
Early pregnancy	1.15 (0.71-1.87)	1.19 (0.73-1.93)

Note: Non-adjusted model: adjustment for none; Adjusted model: adjustment for parental age, BMI status, ethnicity, educational level, physical activity, parity, drinking, smoking, family income, mode of conception, and gestational week; LR, logistic regression; Ref, reference; OR, odds ratio; CI, confidence interval; aOR, adjusted OR; CHD, congenital heart disease

**Table 3:** Associations of FA and/or MV supplementation on fetal CHD in new datasets

FA and/or MV supplementation	Dataset S1		Dataset S2	
	OR (95% CI)	aOR (95% CI)	OR (95% CI)	aOR (95% CI)
None	Ref	Ref	Ref	Ref
Supplementation	1.21 (0.75-1.96)	1.17 (0.72-1.90)	1.17 (0.73-1.90)	1.13 (0.69-1.83)
Pre-pregnancy	1.32 (0.81-2.15)	1.22 (0.75-2.01)	1.28 (0.78-2.09)	1.18 (0.72-1.93)
Early pregnancy	1.13 (0.69-1.83)	1.13 (0.69-1.84)	1.10 (0.67-1.78)	1.09 (0.67-1.78)

Note: Dataset S1 was generated by excluding CHD cases with genetic anomalies that had been labeled in the dataset. Dataset S2 was generated by excluding CHD with other congenital disabilities that had been labeled in the dataset. aOR was estimated by an adjusted LR model, adjusting by parental age, BMI status, ethnicity, educational level, physical activity, parity, drinking, smoking, family income, mode of conception, and gestational week. LR, logistic regression; Ref, reference; OR, odds ratio; CI, confidence interval; aOR, adjusted OR; CHD, congenital heart disease

## Abbreviations

CHD: congenital heart disease
FA: folic acid
MV: multivitamins
RR: relative risk
OR: odds ratios
CI: confidence intervals
aOR: adjusted OR
BMI: body mass index
ID: unique identification
CBCS: the China birth cohort study
WHO: the World Health Organization
Ref: reference
