# Supplementary material for: Association between maternal folic acid and/or multivitamin supplementation time and fetal congenital heart disease: based on the China birth cohort study
Source: Int J Med Sci. 2025 Jan 1;22(2):260–8. doi: 10.7150/ijms.102843 (PMC11704702; doi:10.7150/ijms.102843)
Supplement: Supplementary file 1 — Supplementary figure and table. [file ijmsv22p0260s1.pdf]

Supplementary legends

Fig A1 The rates of fetal CHD in different FA or MV supplementation groups.

Table A1 Associations of FA and/or MV supplementation on fetal CHD in subgroups

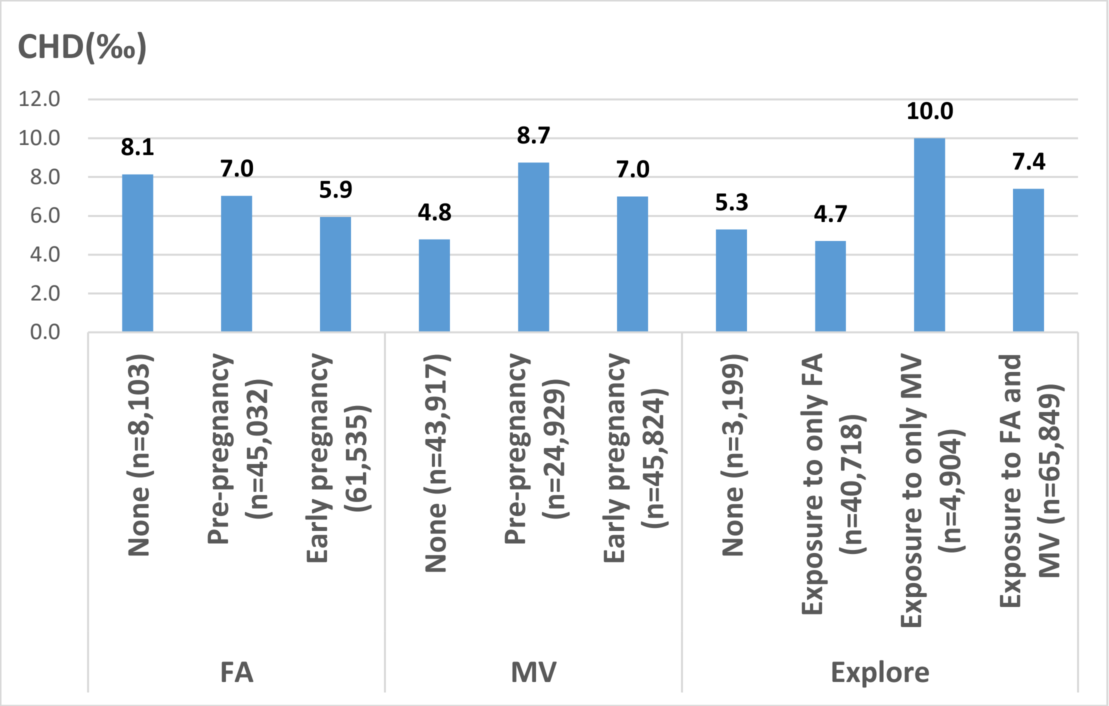

Fig A1 The rates of fetal CHD in different FA or MV supplementation groups.CHD, congenital heart disease; FA, folic acid; MV, multivitamins

Table A1 Associations of FA and/or MV supplementation on fetal CHD in subgroups

Subgroups#			aOR	95%CI	
				Lower	Upper
Family income (CNY/year)	<200,000	Pre-pregnancy	1.21	0.67	2.19
		Early pregnancy	1.03	0.57	1.84
	≥200,000	Pre-pregnancy	1.35	0.55	3.33
		Early pregnancy	1.41	0.58	3.44
Gestational week (weeks)	6~8	Pre-pregnancy	1.55	0.60	3.55
		Early pregnancy	1.56	0.68	3.54
	9~10	Pre-pregnancy	1.98	0.48	8.23
		Early pregnancy	1.68	0.41	6.92
	11~14	Pre-pregnancy	0.86	0.44	1.71
		Early pregnancy	0.76	0.39	1.50
Mode of conception	Natural	Pre-pregnancy	1.23	0.75	2.02
		Early pregnancy	1.15	0.71	1.88
	Assisted	Pre-pregnancy	-	-	-
		Early pregnancy	-	-	-
Maternal age (years)	<35	Pre-pregnancy	1.51	0.80	2.85
		Early pregnancy	1.37	0.73	2.58
	≥35	Pre-pregnancy	0.85	0.38	1.90
		Early pregnancy	0.84	0.39	1.84
Paternal age (years)	<35	Pre-pregnancy	1.29	0.68	2.44
		Early pregnancy	1.24	0.66	2.34
	≥35	Pre-pregnancy	1.20	0.55	2.63
		Early pregnancy	1.01	0.47	2.18
BMI	Normal	Pre-pregnancy	0.89	0.53	1.49
		Early pregnancy	0.84	0.51	1.40
	Low	Pre-pregnancy	3.47	0.47	25.38
		Early pregnancy	2.64	0.36	19.20
	Overweight or Obesity	Pre-pregnancy	3.48	0.47	25.38
		Early pregnancy	2.64	0.36	19.20
Maternal ethnicity	Han	Pre-pregnancy	1.91	0.73	1.95
		Early pregnancy	1.09	0.67	1.78
	Minority	Pre-pregnancy	-	-	-
		Early pregnancy	-	-	-
Maternal educational level	College/university	Pre-pregnancy	1.98	0.81	4.85

		Early pregnancy	1.89	0.78	4.62
	High school or below	Pre-pregnancy	1.64	0.59	4.58
		Early pregnancy	1.32	0.48	3.63
	Postgraduate	Pre-pregnancy	0.60	0.29	1.25
		Early pregnancy	0.57	0.28	1.18
Maternal physical activity	Moderate	Pre-pregnancy	1.34	0.16	11.52
		Early pregnancy	1.97	0.26	15.09
	Light	Pre-pregnancy	1.67	0.82	3.41
		Early pregnancy	1.45	0.72	2.95
	Active	Pre-pregnancy	0.79	0.38	1.63
		Early pregnancy	0.77	0.37	1.57
Paternal physical activity	Moderate	Pre-pregnancy	1.23	0.72	2.12
		Early pregnancy	1.16	0.68	1.99
	Light	Pre-pregnancy	1.01	0.29	3.52
		Early pregnancy	0.38	0.10	1.37
	Active	Pre-pregnancy	-	-	-
		Early pregnancy	-	-	-
Parity	Multipara	Pre-pregnancy	1.35	0.75	2.45
		Early pregnancy	1.21	0.68	2.17
	Nullipara	Pre-pregnancy	1.02	0.42	2.49
		Early pregnancy	0.97	0.40	2.37
Maternal smoking	No	Pre-pregnancy	1.28	0.77	2.13
		Early pregnancy	1.18	0.71	1.95
	Yes	Pre-pregnancy	0.52	0.06	4.74
		Early pregnancy	0.53	0.06	4.49
Paternal smoking	No	Pre-pregnancy	1.94	0.86	4.38
		Early pregnancy	1.69	0.75	3.82
	Yes	Pre-pregnancy	0.83	0.44	1.57
		Early pregnancy	0.84	0.46	1.56
Maternal secondhand smoke exposure	No	Pre-pregnancy	1.34	0.77	2.35
		Early pregnancy	1.25	0.72	2.19
	Yes	Pre-pregnancy	0.97	0.33	2.84
		Early pregnancy	0.81	0.29	2.29
Maternal drinking	No	Pre-pregnancy	1.19	0.73	1.95
		Early pregnancy	1.09	0.67	1.78
	Yes	Pre-pregnancy	-	-	-
		Early pregnancy	-	-	-
Paternal drinking	No	Pre-pregnancy	1.34	71.00	2.54
		Early pregnancy	1.26	0.66	2.37

	Yes	Pre-pregnancy	1.09	1.98	2.39
		Early pregnancy	0.98	0.46	2.12

Note: #The group without maternal exposure to FA and MV was regarded as a reference. CHD, congenital heart disease; FA, folic acid; MV, multivitamins
